# Disparities in the consequences of sarcopenia: implications for African American Veterans

**DOI:** 10.3389/fphys.2014.00250

**Published:** 2014-07-07

**Authors:** Michael O. Harris-Love, Bernadette Adams, Haniel J. Hernandez, Loretta DiPietro, Marc R. Blackman

**Affiliations:** ^1^The Muscle Morphology, Mechanics, and Performance Laboratory, Geriatrics and Extended Care Service, Veterans Affairs Medical CenterWashington, DC, USA; ^2^Research Service, Veterans Affairs Medical CenterWashington, DC, USA; ^3^Department of Exercise Science, School of Public Health and Health Services, The George Washington UniversityWashington, DC, USA; ^4^Physical Therapy, Physical Medicine and Rehabilitation Service, Veterans Affairs Medical CenterWashington, DC, USA; ^5^Department of Medicine, School of Medicine and Health Sciences, The George Washington UniversityWashington, DC, USA

**Keywords:** African Americans, aging, disability, body composition, health disparities, sarcopenia, skeletal muscle, Veterans

## Introduction

The phenomenon of age-related muscle wasting has been long recognized by geriatricians, rehabilitation specialists, and public health practitioners. However, Federal agencies are slow to recognize sarcopenia as a diagnostic term despite the ubiquitous observation of muscle loss as a signature event of aging. The formal adoption of sarcopenia as a clinical diagnosis has been complicated by its evolving operational definition and the varied approaches to the assessment of lean body mass (LBM). Currently, formal assessments of LBM rarely occur within the context of standard geriatric care. The failure to adequately assess muscle tissue within the larger context of geriatric medical care has significant public health implications given the importance of adequate muscle function for “successful aging” and the maintenance of independent living (Visser and Schaap, 2011).

The prevalence of sarcopenia is approximately 25% of the U.S. population over 70 years of age (Baumgartner et al., 1998; Lorenzo, 2009). Additionally, U.S. health care expenditures ascribed to sarcopenia, based on the Third National Health and Nutritional Examination Survey (NHANES III) data, have been conservatively estimated at 11.8 billion dollars (Janssen et al., 2004a). Given the national trends in the Veteran population with respect to age, reported disability levels, and increased representation of African American soldiers, the potential impact of sarcopenia on the Veterans Health Administration (VHA) healthcare system merits consideration.

## African Americans and the VHA healthcare system

The total Veteran population was estimated to be over 22 million in 2012 which also includes approximately 2.2 million women. There was significant minority representation among Veterans during this same period with African Americans members totaling 2.6 million men and 430,000 women. The 2011 Veteran Population Projection Model (VA Office of the Actuary, 2011) projects a nearly 35% decrease in the total Veteran population between 2013 and 2040. Nonetheless, this population trend will be countered by an anticipated concomitant increase in the percentage of minority Veterans from 21 to 34% during this same time period. African Americans will constitute the largest proportion of this projected demographic shift in the Veteran population (National Center for Veterans Analysis and Statistics, 2011). These changes reflect the advent of the All-Voluntary Force and recent military conflicts such as the Gulf War I and II which were notable for the high proportion of participating minority service members with nearly one in three soldiers being African American (Census Bureau, 2010). It is important to note that the Veteran population is older than the general population with a median age of 62 in comparison with a non-Veteran median age of 43. The oldest Veterans reflect a larger proportion of Caucasians (Census Bureau, 2010; National Center for Veterans Analysis and Statistics, 2011) as their median age is 64 in comparison to minority Veterans who have a median age of 54–57. However, the relatively younger subset of minority Veterans coupled with the projected demographic changes suggest that the need to effectively manage geriatric syndromes in African Americans within the VHA healthcare system will continue to increase over time.

## Sarcopenia: diagnostic considerations for African Americans

Sarcopenia is an age-related syndrome characterized by a decrease in muscle mass and associated with a loss of strength and power, diminished functional performance, and increased disability (Newman et al., 2003; Morley et al., 2011). The observation that basic mobility and independent living status may be more dependent on lower extremity muscle performance rather than LBM in older adults (Visser et al., 2000; Kamel, 2003) was the impetus for some investigators to ascribe specific terms (e.g., dynapenia and kratopenia), to characterize the age-related loss of muscle force and power (Morley et al., 2011). Moreover, the Foundation for the National Institutes of Health Sarcopenia Project has proposed to qualify sarcopenia as a form of *skeletal muscle function deficit* to reflect the varied etiology of mobility impairments (Correa-de-Araujo and Hadley, 2014). The recognition of total body mass or body fat as independent factors that influence functional status gave rise to sarcopenic index measures that combine these variables with estimates of LBM (Newman et al., 2003). Consensus groups such as the European Working Group on Sarcopenia in Older People (EWGSOP) (Cruz-Jentoft et al., 2010) have proposed a staged diagnostic criteria for sarcopenia that include elements of LBM, strength, and functional performance in response to these contemporary research findings (Table 1). These consensus groups have recommended customary walking speed as a key component of the sarcopenia screening criteria, and the Society for Sarcopenia, Cachexia and Wasting Disorders (SSCWD) has recommended that only older adults with mobility deficits and/or a history of falls should be considered for sarcopenia screening (Morley et al., 2011). This screening model has potential shortcomings that may be cause for concern. Just as bone fracture is the signal event of osteoporosis, an observed functional limitation may be deemed a signal event of sarcopenia. Moreover, it remains an open question if these aforementioned conditions for sarcopenia screening, derived largely from population-based studies involving relatively healthy community-dwelling adults (Dam et al., 2014), are appropriate for usage among a Veteran population with a high prevalence of comorbid conditions.

**Table 1:** **Categories of the sarcopenia syndrome based on causative factors, body composition, and stages of severity**.

**Causative factors**		**Body composition**		**Staging**	
**Primary Sarcopenia**	Age-related only	**Sarcopenia**	LBM loss criterion only^b^	**Pre- sarcopenia**	LBM loss criterion only^b^
**Secondary Sarcopenia**	• Activity-related	**Sarcopenia class: aLM/h^2^**	A range of criteria based on LBM (relative to stature)^c^	**Sarcopenia**	LBM loss criterion, and strength loss or functional status criteria met^f^
Contributing comorbid factors and behavioral conditions	• Disease-related	• Class I (−1 to −2 SD)			
	• Nutrition-related	• Class II (↓ −2 SD)			
**Myopenia**	LBM loss, affecting functional status or mortality	**Sarcopenia class: SMI**	A range of criteria based on ratio of LBM to body mass (relative to body mass)^d^	**Severe Sarcopenia**	All criteria are met
All-cause designation, independent of age		• Class I (−1 to −2 SD)			
		• Class II (↓ −2 SD)			
**Skeletal Muscle Function Deficit**	Diminished muscle performance^a^, affecting functional status	**Sarcopenic obesity**	LBM maintenance or loss, and body fat criterion met^e^		
All-cause designation, independent of age					

a *Muscle performance as measured by a given measure or estimate of strength, power, or capacity (e.g., endurance/fatigue; Correa-de-Araujo and Hadley, 2014)*.

b *Appendicular lean mass (aLM/h^2^; men and women, respectively): 7.26 and 5.45 kg/m^2^ (Baumgartner et al., 1998); 6.12 and 5.29 kg/m^2^ (African American cohort; Kelly et al., 2009); aLM scaled to body mass index (BMI): 0.789 and 0.512 (aLM_BMI_; men and women, respectively; Dam et al., 2014); use of a single criterion for sarcopenia involving lean body mass (LBM) may include adjustments for height, body fat, and/or body mass by some investigators (Cruz-Jentoft and Morley, 2012)*.

c *Class I: 8.51–10.75 kg/m^2^ in men, and 5.76–6.75 kg/m^2^ in women; Class II: ≤8.50 kg/m^2^ in men, and ≤5.75 kg/m^2^ in women (Janssen et al., 2004a,b)*.

d Class I: 31–37% in men, and 22–28% in women; Class II: ≤30% in men, and ≤21% in women [SMI, skeletal muscle mass index; (muscle mass/body mass) ^*^ 100; Janssen et al., 2002].

e *Body fat percentage (BF%): 27% in men and 38% in women (Baumgartner, 2000); BMI: ≥30 kg/m^2^ (Schrager et al., 2007); cohort specific criteria: 2 highest quintiles of BF% and 2 lowest quintiles of lean body mass (Gomez-Cabello et al., 2011)*.

f *Grip strength: <30 kg in men, and <20 kg in women (Murphy et al., 2013); <26 kg in men, and <16 kg in women (Dam et al., 2014); gait speed: <0.8 m/s (Cruz-Jentoft et al., 2010); gait speed or distance: <1.0 m/s or <400 m during the 6-min walk test (Morley et al., 2011)*.

## Racial/ethnic considerations for body composition assessment

Despite the recent evolution of the diagnostic criteria for sarcopenia, the assessment of LBM remains vital to our understanding and clinical management of age-related muscle dysfunction. A critical issue to consider in addressing possible sarcopenia disparities in African Americans is the influence of racial/ethnic characteristics on common methods of body composition assessment. African Americans have approximately 5–8% higher levels of muscle mass in comparison to Caucasians based on total body potassium assessment (TBK), and this difference applies to both men and women and persists from childhood to old age (Ortiz et al., 1992). African Americans may also have 7–10% greater levels of bone mineral density (BMD) as measured with dual-energy X-ray absorptiometry (DXA), even when controlling for LBM and stature in men (Ettinger et al., 1997). However, the *rate* of age-related LBM and BMD loss may be similar between the two races (Cohn et al., 1977). In addition, the distribution of body fat differs between these groups as Caucasians have greater skinfold thickness in their chest, abdomen, and thighs, whereas African Americans have greater subscapular skinfold thickness (Zillikens and Conway, 1990; Nindl et al., 1998). These phenotypic patterns may confound body composition assessment methods that do not account for these factors. Consequently, an alternative LBM density value of 1.13 g/cm^3^ has been proposed for African Americans instead of the 1.10 g/cm^3^ value typically used in body composition models (Schutte et al., 1984).

The most common whole body tissue composition methods used in sarcopenia research include anthropometry, bioelectrical impedance (BIA), and DXA. Anthropometry is often used in community-health settings and constitutes a broad assessment category that features extremity circumferential measurements, body mass index (BMI), and skin fold measurements. While circumferential measurements have been shown to be associated with diminished activities of daily living (ADL) (Baumgartner et al., 1998), their use as proxy body composition measures systematically underestimate LBM in African Americans based on comparative differences in LBM density, body fat distribution, and even relative body proportions (Wagner and Heyward, 2000). BMI has served as a valuable predictor of critical health outcomes (Kane et al., 2011), but also does not account for racial/ethnic differences in LBM density or body fat distribution and may overestimate the obesity rates in African Americans (Kleerekoper et al., 1994). Skin fold measurements have limitations similar to the aforementioned anthropometric methods, and have also been shown to underestimate LBM density in African Americans (Schutte et al., 1984). In addition, BIA has been used extensively to characterize body composition in population-based studies (Janssen et al., 2002, 2004b; Chien et al., 2008). The interpretation of body composition data involving African Americans and BIA measures may be limited due to assumptions concerning body fat distribution and relative body proportions. However, Wagner et al. (1997) have identified a validated predictive model appropriate for use in this population. Their use of the Segal adiposity-specific equations, with expanded categories per the Stolarczyk modification, yields fairly accurate body composition measures for African Americans, underestimating FFM by 1.8 kg. While lacking the portability of anthropometry and BIA, DXA estimates of muscle mass have a high degree of association with TBK and the imaging method serves as a direct measure of BMD (Wagner and Heyward, 2000; Cruz-Jentoft and Morley, 2012). Therefore, DXA may not be unduly influenced by racial/ethnic differences in LBM density and may serve as an ideal method to assess sarcopenia in studies with multiracial participants and for clinical practice in hospital settings (sarcopenic LBM criterion values, Table 1).

## The prevalence and consequences of sarcopenia in African Americans

Despite the substantial clinical and financial burden attributed to sarcopenia, this geriatric syndrome remains an under-diagnosed condition and is rarely subject to a systematic screening process as a part of customary medical care for older adults (Fielding et al., 2011). The sarcopenia syndrome diminishes functional independence and results in a 3–4 times increased likelihood of developing a disability. In addition, sarcopenia may exacerbate hip fracture incidence due to the loss of requisite strength to maintain dynamic balance (Kamel, 2003). However, while some investigators have shown a significant relationship among diminished LBM, impaired lower extremity strength, functional performance, and disability (Malmstrom et al., 2013), others have reported that a negligible association exists between LBM and disability in community dwelling older adults (Visser et al., 2000). Walking speed, fall risk, negotiating stairs, basic mobility, and independent living status may be more dependent on lower extremity strength and power rather than LBM in older adults (Visser et al., 2000; Kamel, 2003).

The discordance between the age-related loss of muscle mass and diminished function has been observed across ethnic groups. However, this non-intuitive clinical observation is most evident in African Americans. Few studies have reported epidemiological data by ethnic or racial group for sarcopenia. Investigators using data from the Health Aging and Body Composition Study found that 14.3 and 18.4% of African American men and women, respectively, were classified as sarcopenic when adjusting for height and body fat (Newman et al., 2003). Others have noted a prevalence in Caucasians above 25% for men and women across all ages, whereas these values were reported as low as 6–12% in African Americans (Kan, 2009). Nevertheless, studies involving older, community-dwelling, African Americans consistently demonstrate that they have diminished functional performance, and higher levels of dependence with ADLs and risk of disabling conditions, in comparison to other racial/ethnic groups (Miller et al., 2005). What accounts for older African Americans having a higher prevalence of functional limitations and ADL difficulty despite their lower prevalence of sarcopenia?

Both Janssen and Visser have recognized high body fat levels as a confounder in the investigations into the association of LBM with functional limitations and disability (Janssen et al., 2002; Visser et al., 2002). The consideration of body fat and body mass adds an important dimension to the sarcopenia syndrome given that unchanging muscle mass coupled with increased body fat may portend significant functional and metabolic decline (Cruz-Jentoft and Morley, 2012). Moreover, myosteatosis (an increased proportion of intramuscular adipose tissue) has been observed in older adults of African ancestry, and this deleterious adaptation to aging may occur to a greater degree in comparison to Caucasians even when subcutaneous and visceral fat levels are similar among the groups (Song et al., 2004; Miljkovic-Gacic et al., 2008). While the higher risk of disabling conditions observed in African Americans likely involves an array of socioeconomic factors (Fuller-Thomson et al., 2009), high levels of myosteatosis are strongly associated with impaired lower extremity function and may serve as a possible explanation for the departure in linearity between muscle wasting and physical performance (Goodpaster et al., 2001).

Non-contractile features of muscle also affect health outcomes as age-related changes in skeletal muscle may be a critical progenitor of metabolic abnormalities (Albu et al., 2005). While preliminary findings suggest that relative muscle mass may be more strongly associated with insulin resistance than strength (Bijlsma et al., 2013), there are divergent views on this point (Kamel, 2003; Sayer et al., 2005). Data from the NHANES III cohort reveal that sarcopenia is strongly associated with the homeostasis model assessment of insulin resistance and higher HbA1C levels in both non-obese and obese individuals under 60 years of age (Srikanthan et al., 2010). However, the relationship between sarcopenia and a higher prevalence of type 2 diabetes was only observed in obese individuals. Miljkovic et al. (2009) determined that Afro-Caribbean men had higher myosteatosis levels and a lower body fat percentage in comparison to Caucasian men. Myosteatosis was associated with type 2 diabetes in both groups, but 44% of the Afro-Caribbean men were diagnosed with the metabolic disorder in comparison to 13% of the Caucasian men. While the negative impact of low muscle mass on glucose homeostasis cannot be discounted (Bijlsma et al., 2013), excessive intramuscular adipose tissue and intramyocellular lipid content may disrupt insulin receptor activity, limit glucose transport, and potentially result in adipokine release from local concentrations of adipose tissue (Miljkovic et al., 2009). The case could be made to incorporate age-related changes in muscle quality into the broad view of the sarcopenia diagnosis. However, more prospective study will be needed to better understand the role of myosteatosis in the pathogenesis of diminished insulin sensitivity and diabetes. Given that African Americans are 1.6–1.7 times as likely to have diabetes in comparison to Caucasians within the VA and civilian population, it may be a compelling interest of the Federal health agencies, professional and scientific organizations, and other stakeholders to study the impact of age-related muscle changes and how they interact with racial/ethnic group factors in health disparities (National Center for Veterans Analysis and Statistics, 2011).

## Funding source

This publication was partially supported by VISN 5 Pilot Research Grant (VISN 5; VA Station: 688), VHA/VA Capitol Health Care Network. Additional support was provided by the VA Office of Academic Affiliations (OAA; 38 U.S.C 7406) and the VA Office of Research and Development.

### Conflict of interest statement

The authors declare that the research was conducted in the absence of any commercial or financial relationships that could be construed as a potential conflict of interest.

## References

[B1] Albu J. B. Kovera A. J. Allen L. Wainwright M. Berk E. Raja-Khan N. (2005). Independent association of insulin resistance with larger amounts of intermuscular adipose tissue and a greater acute insulin response to glucose in African American than in white nondiabetic women. Am. J. Clin. Nutr. 82, 1210–1217 16332653 10.1093/ajcn/82.6.1210 PMC2670467

[B2] Baumgartner R. N. (2000). Body composition in healthy aging. Ann. N.Y. Acad. Sci. 904, 437–448 10.1111/j.1749-6632.2000.tb06498.x 10865787

[B3] Baumgartner R. N. Koehler K. M. Gallagher D. Romero L. Heymsfield S. B. Ross R. R. (1998). Epidemiology of sarcopenia among the elderly in New Mexico. Am. J. Epidemiol. 147, 755–763 10.1093/oxfordjournals.aje.a009520 9554417

[B4] Bijlsma A. Y. Meskers C. G. M. van Heemst D. Westendorp R. G. J. de Craen A. J. M. Maier A. B. (2013). Diagnostic criteria for sarcopenia relate differently to insulin resistance. Age (Dordr). 35, 2367–2375 10.1007/s11357-013-9516-0 23407994 PMC3824998

[B5] Census Bureau. (2010). Public Use Microdata Areas (PUMAs) [Internet]. Washington, DC: Census Bureau Available online at: http://www.census.gov/geo/reference/puma.html

[B6] Chien M.-Y. Huang T.-Y. Wu Y.-T. (2008). Prevalence of sarcopenia estimated using a bioelectrical impedance analysis prediction equation in community-dwelling elderly people in Taiwan. J. Am. Geriatr. Soc. 56, 1710–1715 10.1111/j.1532-5415.2008.01854.x 18691288

[B7] Cohn S. H. Abesamis C. Zanzi I. Aloia J. F. Yasumura S. Ellis K. J. (1977). Body elemental composition: comparison between black and white adults. Am. J. Physiol. 232, E419–E422 851185 10.1152/ajpendo.1977.232.4.E419

[B8] Correa-de-Araujo R. Hadley E. (2014). Skeletal muscle function deficit: a new terminology to embrace the evolving concepts of sarcopenia and age-related muscle dysfunction. J. Gerontol. A Biol. Sci. Med. Sci. 69, 591–594 10.1093/gerona/glt208 24737562 PMC3999854

[B9] Cruz-Jentoft A. J. Baeyens J. P. Bauer J. M. Boirie Y. Cederholm T. Landi F. (2010). Sarcopenia: European consensus on definition and diagnosis: report of the European Working Group on Sarcopenia in Older People. Age Ageing 39, 412–423 10.1093/ageing/afq034 20392703 PMC2886201

[B10] Cruz-Jentoft A. J. Morley J. E. (eds.). (2012). Sarcopenia. Chichester: John Wiley and Sons

[B11] Dam T.-T. Peters K. W. Fragala M. Cawthon P. M. Harris T. B. McLean R. (2014). An evidence-based comparison of operational criteria for the presence of sarcopenia. J. Gerontol. A Biol. Sci. Med. Sci. 69, 584–590 10.1093/gerona/glu013 24737561 PMC3991139

[B12] Ettinger B. Sidney S. Cummings S. R. Libanati C. Bikle D. D. Tekawa I. S. (1997). Racial differences in bone density between young adult black and white subjects persist after adjustment for anthropometric, lifestyle, and biochemical differences. J. Clin. Endocrinol. Metab. 82, 429–434 10.1210/jcem.82.2.3732 9024231

[B13] Fielding R. A. Vellas B. Evans W. J. Bhasin S. Morley J. E. Newman A. B. (2011). Sarcopenia: an undiagnosed condition in older adults. Current consensus definition: prevalence, etiology, and consequences. International working group on sarcopenia. J. Am. Med. Dir. Assoc. 12, 249–256 10.1016/j.jamda.2011.01.003 21527165 PMC3377163

[B14] Fuller-Thomson E. Nuru-Jeter A. Minkler M. Guralnik J. M. (2009). Black-White disparities in disability among older Americans: further untangling the role of race and socioeconomic status. J. Aging Health 21, 677–698 10.1177/0898264309338296 19584411 PMC2907066

[B15] Gomez-Cabello A. Pedrero-Chamizo R. Olivares P. R. Luzardo L. Juez-Bengoechea A. Mata E. (2011). Prevalence of overweight and obesity in non-institutionalized people aged 65 or over from Spain: the elderly EXERNET multi-centre study. Obes. Rev. 12, 583–592 10.1111/j.1467-789X.2011.00878.x 21535360

[B16] Goodpaster B. H. Carlson C. L. Visser M. Kelley D. E. Scherzinger A. Harris T. B. (2001). Attenuation of skeletal muscle and strength in the elderly: the health ABC study. J. Appl. Physiol. (1985) 90, 2157–2165 Available online at: http://jap.physiology.org/cgi/pmidlookup?view=long&pmid=11356778 11356778 10.1152/jappl.2001.90.6.2157

[B17] Janssen I. Baumgartner R. N. Ross R. Rosenberg I. H. Roubenoff R. (2004b). Skeletal muscle cutpoints associated with elevated physical disability risk in older men and women. Am. J. Epidemiol. 159, 413–421 10.1093/aje/kwh058 14769646

[B18] Janssen I. Heymsfield S. B. Ross R. (2002). Low relative skeletal muscle mass (sarcopenia) in older persons is associated with functional impairment and physical disability. J. Am. Geriatr. Soc. 50, 2074–2079 10.1046/j.1532-5415.2002.50216.x 12028177

[B19] Janssen I. Shepard D. S. Katzmarzyk P. T. Roubenoff R. (2004a). The healthcare costs of sarcopenia in the United States. J. Am. Geriatr. Soc. 52, 80–85 10.1111/j.1532-5415.2004.52014.x 14687319

[B20] Kamel H. K. (2003). Sarcopenia and aging. Nutr. Rev. 61(5 pt 1), 157–167 10.1301/nr.2003.may.157-167 12822704

[B21] Kan G. A. V. (2009). Epidemiology and consequences of sarcopenia. J. Nutr. Health Aging 13, 708–712 10.1007/s12603-009-0201-z 19657554 PMC12275513

[B22] Kane R. L. Talley K. M. Shamliyan T. Pacala J. T. (2011). Common Syndromes in Older Adults Related to Primary and Secondary Prevention [Internet]. Rockville, MD: Agency for Healthcare Research and Quality (US) [cited 2013 Nov 19]. Available online at: http://www.ncbi.nlm.nih.gov/books/NBK62074/21901865

[B23] Kelly T. L. Wilson K. E. Heymsfield S. B. (2009). Dual energy X-Ray absorptiometry body composition reference values from NHANES. PLoS ONE 4:e7038 10.1371/journal.pone.0007038 19753111 PMC2737140

[B24] Kleerekoper M. Nelson D. A. Peterson E. L. Wilson P. S. Jacobsen G. Longcope C. (1994). Body composition and gonadal steroids in older white and black women. J. Clin. Endocrinol. Metab. 79, 775–779 8077360 10.1210/jcem.79.3.8077360

[B25] Lorenzo C. (2009). Body composition and physical function in older adults. Obesity 17, 211–212 10.1038/oby.2008.506 19169220

[B26] Malmstrom T. K. Miller D. K. Herning M. M. Morley J. E. (2013). Low appendicular skeletal muscle mass (ASM) with limited mobility and poor health outcomes in middle-aged African Americans. J. Cachexia Sarcopenia Muscle 4, 179–186 10.1007/s13539-013-0106-x 23532635 PMC3774914

[B27] Miljkovic I. Cauley J. A. Petit M. A. Ensrud K. E. Strotmeyer E. Sheu Y. (2009). Greater adipose tissue infiltration in skeletal muscle among older men of African ancestry. J. Clin. Endocrinol. Metab. 94, 2735–2742 10.1210/jc.2008-2541 19454588 PMC2730872

[B28] Miljkovic-Gacic I. Gordon C. L. Goodpaster B. H. Bunker C. H. Patrick A. L. Kuller L. H. (2008). Adipose tissue infiltration in skeletal muscle: age patterns and association with diabetes among men of African ancestry. Am. J. Clin. Nutr. 87, 1590–1595 18541544 10.1093/ajcn/87.6.1590 PMC2532786

[B29] Miller D. K. Wolinsky F. D. Malmstrom T. K. Andresen E. M. Miller J. P. (2005). Inner city, middle-aged African Americans have excess frank and subclinical disability. J. Gerontol. A Biol. Sci. Med. Sci. 60, 207–212 10.1093/gerona/60.2.207 15814864

[B30] Morley J. E. Abbatecola A. M. Argiles J. M. Baracos V. Bauer J. Bhasin S. (2011). Sarcopenia with limited mobility: an international consensus. J. Am. Med. Dir. Assoc. 12, 403–409 10.1016/j.jamda.2011.04.014 21640657 PMC5100674

[B31] Murphy R. A. Ip E. H. Zhang Q. Boudreau R. M. Cawthon P. M. Newman A. B. (2013). Transition to sarcopenia and determinants of transitions in older adults: a population-based study. J. Gerontol. A Biol. Sci. Med. Sci. 69, 751–758 10.1093/gerona/glt131 24013673 PMC4022098

[B32] National Center for Veterans Analysis and Statistics. (2011). Profile of Veterans: 2009 [Internet]. Washington, DC: NCVAS Available online at: http://www.va.gov/vetdata/docs/SpecialReports/Profile_of_Veterans_2009_FINAL.pdf

[B33] Newman A. B. Kupelian V. Visser M. Simonsick E. Goodpaster B. Nevitt M. (2003). Sarcopenia: alternative definitions and associations with lower extremity function. J. Am. Geriatr. Soc. 51, 1602–1609 10.1046/j.1532-5415.2003.51534.x 14687390

[B34] Nindl B. C. Kraemer W. J. Emmert W. H. Mazzetti S. A. Gotshalk L. A. Putukian M. (1998). Comparison of body composition assessment among lean black and white male collegiate athletes. Med. Sci. Sports Exerc. 30, 769–776 10.1097/00005768-199805000-00020 9588622

[B35] Ortiz O. Russell M. Daley T. L. Baumgartner R. N. Waki M. Lichtman S. (1992). Differences in skeletal muscle and bone mineral mass between black and white females and their relevance to estimates of body composition. Am. J. Clin. Nutr. 55, 8–13 1728823 10.1093/ajcn/55.1.8

[B36] Sayer A. A. Dennison E. M. Syddall H. E. Gilbody H. J. Phillips D. I. W. Cooper C. (2005). Type 2 diabetes, muscle strength, and impaired physical function: the tip of the iceberg? Diabetes Care 28, 2541–2542 10.2337/diacare.28.10.2541 16186295

[B37] Schrager M. A. Metter E. J. Simonsick E. Ble A. Bandinelli S. Lauretani F. (2007). Sarcopenic obesity and inflammation in the InCHIANTI study. J. Appl. Physiol. 102, 919–925 10.1152/japplphysiol.00627.2006 17095641 PMC2645665

[B38] Schutte J. E. Townsend E. J. Hugg J. Shoup R. F. Malina R. M. Blomqvist C. G. (1984). Density of lean body mass is greater in blacks than in whites. J. Appl. Physiol. 56, 1647–1649 6735823 10.1152/jappl.1984.56.6.1647

[B39] Song M.-Y. Ruts E. Kim J. Janumala I. Heymsfield S. Gallagher D. (2004). Sarcopenia and increased adipose tissue infiltration of muscle in elderly African American women. Am. J. Clin. Nutr. 79, 874–880 Available online at: http://www.ajcn.org/cgi/pmidlookup?view=long&pmid=15113728 15113728 10.1093/ajcn/79.5.874

[B40] Srikanthan P. Hevener A. L. Karlamangla A. S. (2010). Sarcopenia exacerbates obesity-associated insulin resistance and dysglycemia: findings from the National Health and Nutrition Examination Survey III. PLoS ONE 5:e10805 10.1371/journal.pone.0010805 22421977 PMC3279294

[B41] VA Office of the Actuary. (2011). The Veteran Population Projection Model 2011. Washington, DC: OACT

[B42] Visser M. Kritchevsky S. B. Goodpaster B. H. Newman A. B. Nevitt M. Stamm E. (2002). Leg muscle mass and composition in relation to lower extremity performance in men and women aged 70 to 79: the health, aging and body composition study. J. Am. Geriatr. Soc. 50, 897–904 10.1046/j.1532-5415.2002.50217.x 12028178

[B43] Visser M. Newman A. B. Nevitt M. C. Kritchevsky S. B. Stamm E. B. Goodpaster B. H. (2000). Reexamining the sarcopenia hypothesis - muscle mass versus muscle strength. Ann. N.Y. Acad. Sci. 904, 456–461 10.1111/j.1749-6632.2000.tb06500.x 10865789

[B44] Visser M. Schaap L. A. (2011). Consequences of sarcopenia. Clin. Geriatr. Med. 27, 387–399 10.1016/j.cger.2011.03.006 21824554

[B45] Wagner D. R. Heyward V. H. (2000). Measures of body composition in blacks and whites: a comparative review. Am. J. Clin. Nutr. 71, 1392–1402 Available online at: http://www.ajcn.org/cgi/pmidlookup?view=long&pmid=10837277 10837277 10.1093/ajcn/71.6.1392

[B46] Wagner D. R. Heyward V. H. Kocina P. S. Stolarczyk L. M. Wilson W. L. (1997). Predictive accuracy of BIA equations for estimating fat-free mass of black men. Med. Sci. Sports Exerc. 29, 969–974 10.1097/00005768-199707000-00018 9243498

[B47] Zillikens M. C. Conway J. M. (1990). Anthropometry in blacks: applicability of generalized skinfold equations and differences in fat patterning between blacks and whites. Am. J. Clin. Nutr. 52, 45–51 2360551 10.1093/ajcn/52.1.45

